# Tumor Necrosis Factor Family Member Profile Predicts Prognosis and Adjuvant Chemotherapy Benefit for Patients With Small-Cell Lung Cancer

**DOI:** 10.3389/fimmu.2021.745769

**Published:** 2021-11-18

**Authors:** Zhihui Zhang, Peng Wu, Chaoqi Zhang, Yuejun Luo, Guochao Zhang, Qingpeng Zeng, Lide Wang, Zhaoyang Yang, Nan Sun, Jie He

**Affiliations:** ^1^ Department of Thoracic Surgery, National Cancer Center/National Clinical Research Center for Cancer/Cancer Hospital, Chinese Academy of Medical Sciences and Peking Union Medical College, Beijing, China; ^2^ Department of Pathology, National Cancer Center/National Clinical Research Center for Cancer/Cancer Hospital, Chinese Academy of Medical Sciences and Peking Union Medical College, Beijing, China

**Keywords:** small cell lung cancer, antitumor immunity, tumor necrosis factor family, prognostic prediction, chemotherapy response

## Abstract

Tumor necrosis factor (TNF) family members participate in the body’s antitumor immunity response and influence tumor prognosis and treatment response. However, little is known about the roles of TNF family members in small cell lung cancer (SCLC). Therefore, we conducted the first comprehensive investigation of TNF family members in patients with SCLC, with the goal of using them to predict prognosis and chemotherapy benefit. Abnormal genetic alterations and expression of TNF family members were found to be widespread in SCLC patients. Using LASSO Cox regression analysis, we constructed a TNF family-based signature that separated SCLC patients in the training set (n=77) into high- and low-risk groups with distinct survival and chemotherapy benefit, and the signature was well-validated in the validation set (n=137) by RT-qPCR. Importantly, the signature exhibited superior predictive performance and was identified as a novel independent prognostic factor. Additionally, different immune phenotypes were found between the low-risk and high-risk groups, and high-risk patients had higher CMTM6 expression, suggesting that these patients could benefit from therapeutic methods targeting CMTM6. We constructed the first clinically applicable TNF family-based signature for predicting prognosis and chemotherapy benefit for patients with SCLC. The findings reported here provide a new method for predicting the prognosis of SCLC patients and optimizing clinical management.

## Introduction

Small cell lung cancer (SCLC) is a recalcitrant malignancy that accounts for 13–15% of all lung cancers (1). It is an aggressive neuroendocrine tumor characterized by rapid growth and early and widespread hematogenous metastases (2). Patients with SCLC generally receive dismal prognosis, with an average 5-year overall survival (OS) of less than 5%, and median survival of only 7–12 months (3). Most SCLC patients are diagnosed in the advanced stage and are thus not candidates for surgery. Chemotherapy has been the standard treatment for first- and second-line management of SCLC for more than three decades (4, 5). However, patients often rapidly develop drug resistance or relapse after remission, even if they are initially sensitive to chemotherapy (6). The scarcity of SCLC tissue samples has hindered the process of investigating the mechanism underlying its rapid progression and treatment resistance and identifying tumor-specific prognosis predictive biomarkers in large-scale studies. Therefore, there is an urgent and unmet need to identify biomarkers that can be used to accurately predict prognosis and chemotherapy benefit so that clinical management of SCLC patients can be improved.

Immunotherapy has revolutionized cancer treatment over the past decade and has extended the life expectancy of patients with malignant tumors, including SCLC (7, 8). It has been reported that a high mutational burden is closely related to immunotherapy efficacy (9). SCLC is characterized by a high mutational burden, and these mutations can give rise to immunogenic neoantigens, which can be presented by the major histocompatibility complex and recognized by T cells, leading to tumor-specific CD8+ T cell activity (10). In addition, a highly activated host tumor immune microenvironment (TIME) is closely associated with better clinical outcomes for SCLC patients, indicating that immunotherapy could be a promising treatment for SCLC (11). In recent years, immunotherapy has achieved some encouraging results in SCLC patients. The combination of PD-L1 inhibitors like atezolizumab and durvalumab with chemotherapy has enhanced the OS of SCLC patients by 2–3 months since they were approved as first-line treatments for SCLC by the Food and Drug Administration (FDA) (12, 13). However, therapeutic interventions targeting the two best-described B7-CD28 family immunotherapy targets—T-lymphocyte protein 4 (CTLA-4) and programmed cell death protein-1 (PD-1)—have failed to achieve favorable results in patients with SCLC (14–16).Therefore, a better understanding of the TIME and immune checkpoint blockades has the potential to lead to promising new SCLC treatments. The immune molecular expression profile in the TIME is associated with conventional chemotherapy efficacy (17). Tumors influence the TIME by suppressing extracellular signals; the immunologic milieu can also affect malignant behavior and progression (18). Our previous study confirmed that TIME-related biomarkers could be used to effectively predict radiotherapy and chemotherapy benefit in ESCC patients (19). Hence, understanding the complex and diverse molecular profile of the immunologic genome in the TIME is also indispensable for maximizing the benefits of SCLC therapy.

Tumor necrosis factor (TNF) family is a master mediator of survival signaling in inflammation, which plays an important role at many stages of the immune response (20). The TNF superfamily (TNFSF) and TNF receptor superfamily (TNFRSF) include nineteen ligands and twenty-nine receptors, which play important roles in modulating cellular functions. T cell-related diseases may be treated by manipulating ligand-receptor interactions involved in inflammatory and autoimmune diseases (21). TNFSF is a type II transmembrane protein featuring a TNF homology domain. TNFRSF members can stimulate T-cell responsiveness and define niches for T cell memory (21, 22). Several TNFRSF members have been identified as emerging cancer immunotherapy targets (23–25). Unfortunately, the expression profile and clinical relevance of the TNF family in SCLC remain poorly understood.

We focused on TNF family members and sought to determine their expression profiles and clinical significance to SCLC. Survival and multivariate analyses were used to identify the genes with the greatest prognostic value. We constructed a predictive model based on TNF family members for patients with SCLC and used information extracted from public datasets to validate our novel signature in different cohorts and clinical subsets. Finally, we determined the predictive value of immune markers for adjuvant chemotherapy (ACT) in patients with SCLC. To the best of our knowledge, this signature is the first prognostic indicator based on the TNF family for SCLC. Our results may help define new therapeutic strategies, optimize the application of precision medicine, and offer renewed hope for patients with SCLC.

## Materials and Methods

### Public mRNA Expression Datasets and Clinical Information

We compiled comprehensive genomic profiles with complete clinical parameters and survival information for SCLC patients included within a training cohort (https://www.cbioportal.org/study/summary?id=sclc_ucologne_2015) (26). Microarray dataset GSE40275 was used to identify genes with different expression levels between SCLC and matching normal lung tissues (downloaded from the Gene Expression Omnibus (GEO) at https://www.ncbi.nlm.nih.gov/geo/). For the validation cohort, samples from 137 patients with biopsy-proven SCLC tissues were obtained from the National Cancer Center (NCC). The samples included archived formalin-fixed paraffin-embedded (FFPE) blocks and were originally collected from patients from January 2009 to November 2018. All patients had primary tumors at stages I–IV, and the diagnoses were independently reviewed by two expert pathologists from the NCC. The protocol of this study was approved by the Ethics Committee of the National Cancer Center/Cancer Hospital of the Chinese Academy of Medical Sciences. All participants provided written informed consent prior to completion of any study-related procedures. Detailed information about the samples is listed in **Table 1**.

**Table 1:** Clinicopathological characteristics of enrolled patients.

Characteristics	Training Cohort (*N* = 77)	Validation Cohort (*N* = 137)
Sex		
Male	54 (70.13%)	105 (76.64%)
Female	23 (29.87%)	32 (23.36%)
Age, years		
≥60	57 (74.03%)	65 (47.45%)
<60	20 (25.97%)	72 (52.55%)
Smoking history		
Yes	72 (96.00%)	86 (62.78%)
No	3 (4.00%)	51 (37.23%)
SCLC staging		
I	33 (42.85%)	46 (33.58%)
II	14 (18.18%)	45 (32.85%)
III	21 (27.27%)	46 (33.58%)
IV	9 (11.69%)	0 (0.00%)
OS state		
Alive	29 (37.66%)	59 (43.07%)
Death	48 (62.34%)	78 (56.93%)

SCLC, small cell lung cancer; OS, overall survival.

### RNA Extraction, cDNA Synthesis, and Quantitative Reverse-Transcriptase Polymerase Chain Reaction (qRT-PCR) Validation

Total RNA was extracted from SCLC tissue samples using the RNAiso Plus reagent (Takara, #9109) according to the manufacturer’s protocol. cDNA was synthesized from total RNA using the FastKing RT Kit (with gDNase) (Tiangen, KR116) in two steps. qRT-PCT was performed using the QuantiNova SYBR Green PCR Kit (Qiagen, 208054) with a reaction mixture containing 1 μl complementary DNA, 5 μl SYBR Green, 1.4 μl PCR primers (0.7 μl forward primer and 0.7 μl reverse primer), 1 μl QN ROX Reference Dye, and 1.6 μl RNase-Free water, totaling 10 μl in volume. GAPDH was selected as a control gene. Detailed sequences of the primer pairs are listed in **Table S2**. All data were log2-transformed, and expression levels were calculated using the 2^-ΔΔCt^ method.

### Functional Enrichment Analyses

To further explore the biological functions of TNF family-related genes, Gene Ontology (GO) and Kyoto Encyclopedia of Genes and Genomes (KEGG) analyses were carried out using DAVID 6.8 (http://david.abcc.ncifcrf.gov).

### Gene Set Enrichment Analysis (GSEA)

GSEA was performed to interpret high-dimensional gene expression data (http://www.broadinstitute.org/gsea) (27). The enrichment score was the maximum distance from the middle of the ranking used to test gene set significance. An FDR<0.05 was regarded as indicating significant enrichment.

### Gene Set Variation Analysis (GSVA)

GSVA is a non-parametric unsupervised method that allows gene set enrichment of expression data and gene sets in each sample (28). GSVA was implemented with the GSVA package (http://www.bioconductor.org) under R software version 3.5.1 to reveal the biological activity of selected signaling pathways over a sample population.

### Estimate of Immune-Microenvironment and Tumor-Infiltrating Immune Cells

We used the ESTIMATE algorithm to infer the constituent cellular fraction, and calculated stromal and immune scores to assess tumor tissue purity (29). The proportion of immune cells in the TIME was quantitatively estimated by CIBERSORT (http://cibersort.stanford.edu/) based on gene expression profiles available from a public dataset. The LM22 signature algorithm containing 547 genes was used to distinguish 22 types of immune cells.

### Statistical Analysis

Social demographics and clinicopathological features are presented as mean ± standard deviation or median (interquartile range) for continual variables and frequency (n) and proportion (%) for categorical variables. We used Kaplan-Meier survival analysis and least absolute shrinkage and selection operator (LASSO) Cox regression analysis to identify molecules of the TNF family with high prognostic value. The signature and risk formula were constructed using the selected genes through linear combination of gene expression levels. Patients were separated into high- or low-risk groups based on the optimal cutoff point. In the survival analysis, relapse-free survival (RFS) and OS were calculated using the Kaplan-Meier method, and between-group differences were compared using the log-rank test. Receiver operating characteristic (ROC) curves were used to show the predictive accuracy of the gene signature, and the performance of the model was determined based on the values of the area under the curve (AUC) and concordance index (C-index). Univariate and multivariable Cox proportional hazards regression models were used to determine whether the signature could independently predict the clinical outcomes of patients with SCLC after controlling for potential confounders. The relationship between TNF family molecules and immune checkpoints was also evaluated using a correlational analysis, in which correlation coefficients, CIs, and P values were generated. All statistical analyses were performed using R 3.5.1, and a two-tail P value <0.05 was considered statistically significant.

## Results

### The Expression Profiles of TNF Family Genes Display Significant Differences Between SCLC and Adjacent Normal Tissues

**Figure 1:**
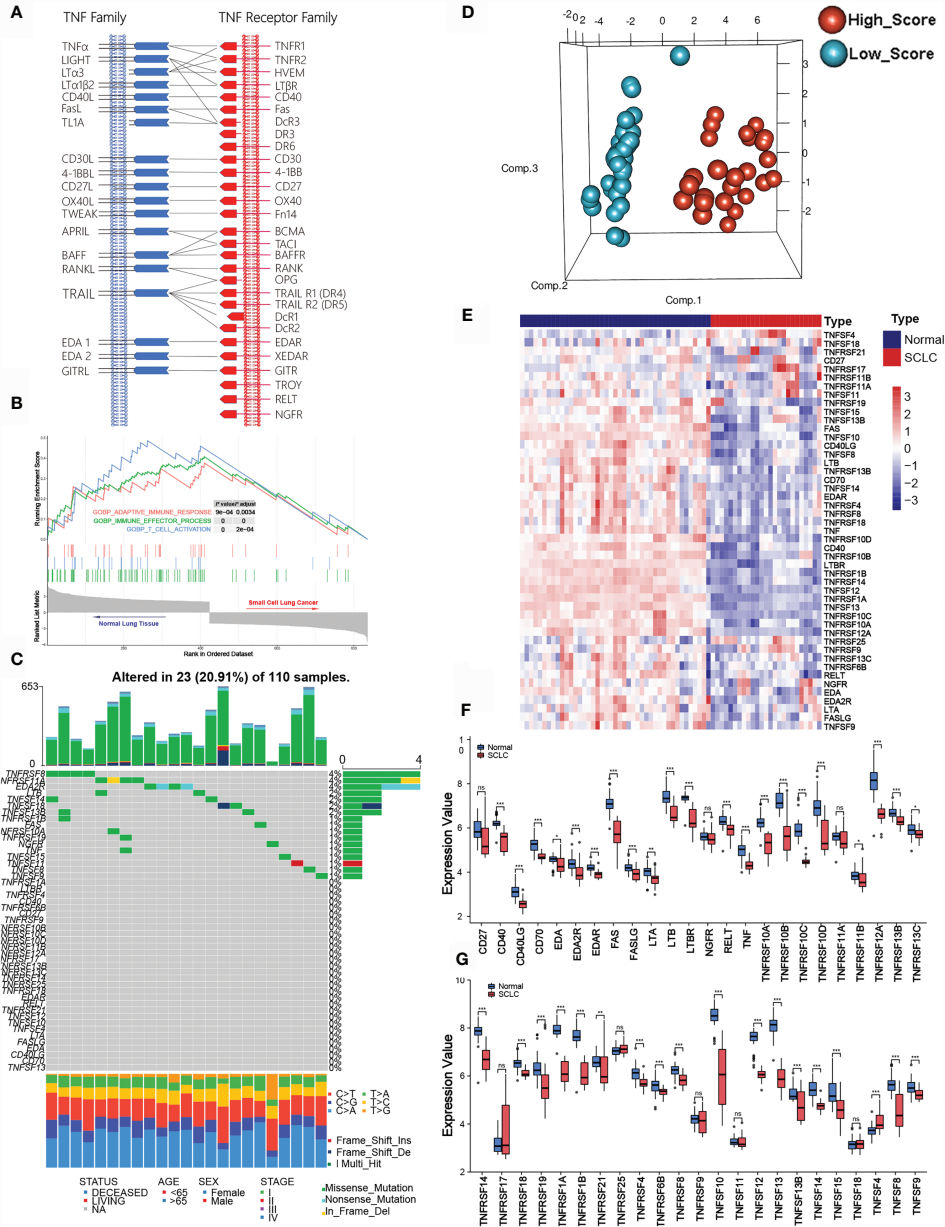
Molecular characteristic and expression profile of tumor necrosis factor (TNF) family members in small cell lung cancer. **(A)** Interaction between the ligands and receptors of the TNF family. Ligands and receptors are shown at the left (blue) and right (red) in the diagram. The vertical lines represent the cytoplasmic membrane of corresponding cells, and the horizontal lines with arrows represent ligand-receptor pairs. **(B)** GSEA of normal lung tissues and SCLC tissues. **(C)** The mutation landscape waterfall plot of TNFSF/TNFRSF members in 110 patients with SCLC from the training cohort. Each bar represents the mutation for each sample. **(D)** Principal component analysis (PCA) based on the expression levels of TNF family members between adjacent normal tissue and tumor tissue in the training set. **(E)** The expression details of TNF family members between SCLC samples and normal lung tissue. **(F, G)** Boxplots show differences in the expression values of TNF family members. *p < 0.05; **p < 0.01; ***p < 0.00; ns, no significance.

According to previous literature, we systematically explored the landscape of TNFSF/TNFRSF; 19 ligands and 29 receptors were included in our study. The interactions between ligands and receptors are summarized in **Figure 1A**. Firstly, we conducted a full assessment of different biological processes based on differentially expressed genes between SCLC samples and adjacent normal lung samples using GSEA. The results showed that the immune response was depressed in SCLC samples, especially T cell activation (**Figure 1B**). This result suggests that TNF family members—one of the most significant regulator families in T cell activation—may exhibit remarkable abnormality in SCLC patients. Next, we visualized the somatic mutation profile landscape of TNFSF/TNFRSF members in SCLC patients (**Figure 1C**). As depicted by the waterfall plot, we observed a mutation frequency of 20.91% (23/110), with non-synonymous mutations being the most common.

Next, we investigated whether the expression levels of TNF family molecules could be used to distinguish SCLC and normal tissue samples by principal component analysis (PCA), and our results indicated significant between-group heterogeneity (**Figure 1D**). Moreover, the heatmap showed low expression of the TNF family in SCLC samples (**Figure 1E**); detailed expression level information is displayed in the boxplots (**Figures 1F, G**). Additionally, we used Pearson correlation analysis to explore the relationships between TNFSF/TNFRSF members (**Figure S1**). Nearly all of the selected molecules were positively associated with each other, with the exceptions of TNFRSF25, TNFRSF11A, and TNFRSF19.

### Identification of Prognostic TNF Family Genes in SCLC Patients

**Figure 2:**
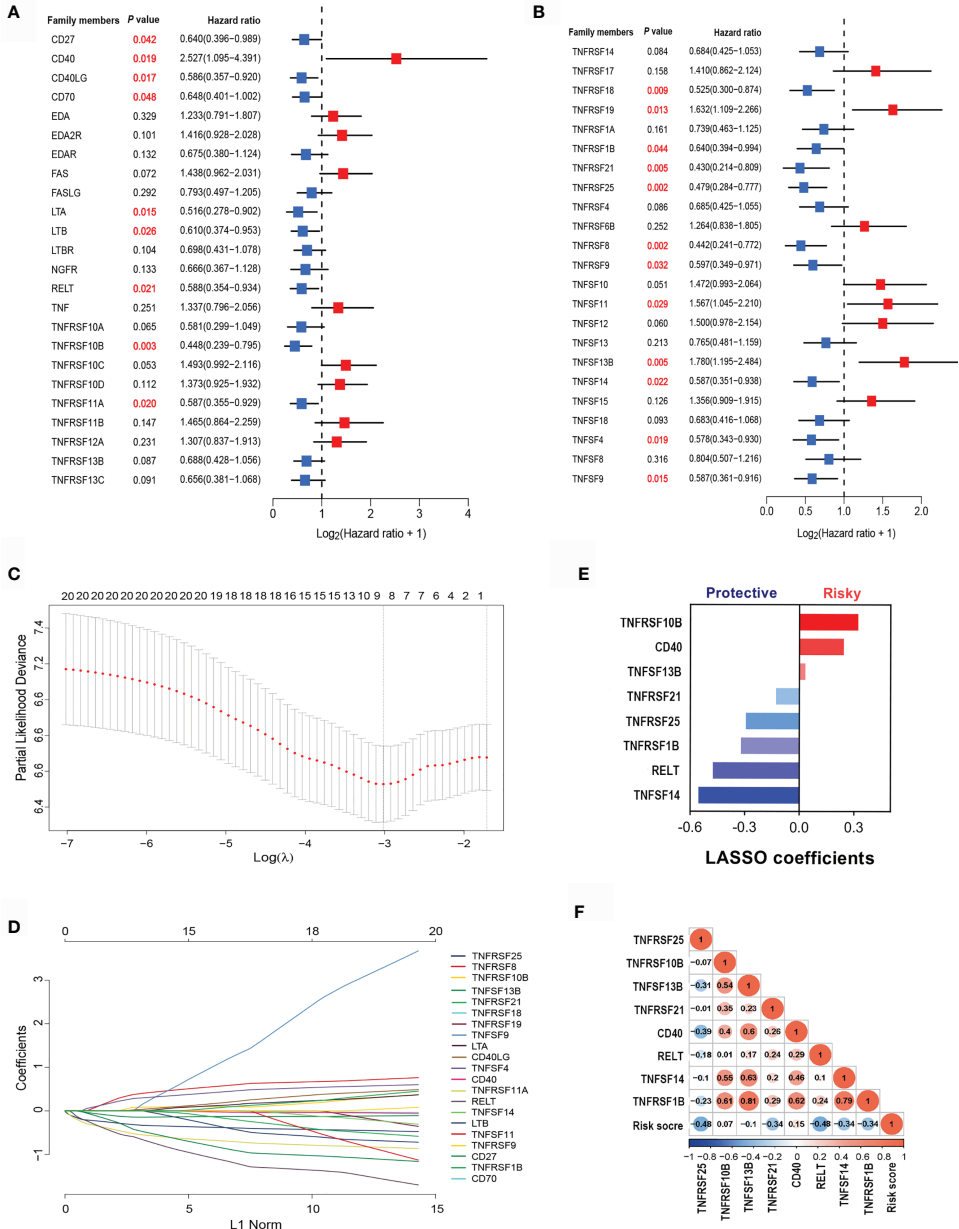
The clinical significance of TNF family members in SCLC patients. **(A, B)** The forest plots of hazard ratios (HRs) show the prognostic values of TNF members. **(C)** Tuning parameter (λ) selection cross-validation (n = 100) error curve for relevant prognostic genes. Red dots represent the partial likelihood deviance values, and grey lines represent the standard error (SE). The vertical lines are the optimal values according to the minimum and the 1-SE criteria. **(D)** The LASSO coefficient profiles of the most useful prognostic genes from the TNF family. **(E)** The weighting coefficient values for each prognostic gene. **(F)** Correlation matrix of the risk score and eight selected genes.

To identify clinically significant TNF family genes, we analyzed all TNFSF/TNFRSF members and selected 21 genes that were statistically associated with prognosis for the training cohort (**Figures 2A, B** and **Table S1**). Next, we used a LASSO Cox regression model to reduce dimensionality and identify the TNF family members with the greatest prognostic value. TNFRSF10B, CD40, TNFSF13B, TNFRSF21, TNFRSF25, TNFRSF1B, RELT, and TNFSF14 were selected by the model and used to construct a TNF family-based signature (**Figures 2C, D**). The weighting coefficients are presented in **Figure 2E**. The results indicated that TNFRSF10B, CD40, and TNFSF13B were risk factors for SCLC patients, while TNFRSF21, TNFRSF25, TNFRSF1B, RELT, and TNFSF14 were protective prognostic factors. The relationships between the TNF family-based signature and the eight genes are presented in **Figure 2F**.

### Construction and Validation of the TNF Family Based-Signature With a Training Cohort of SCLC Patients

**Figure 3:**
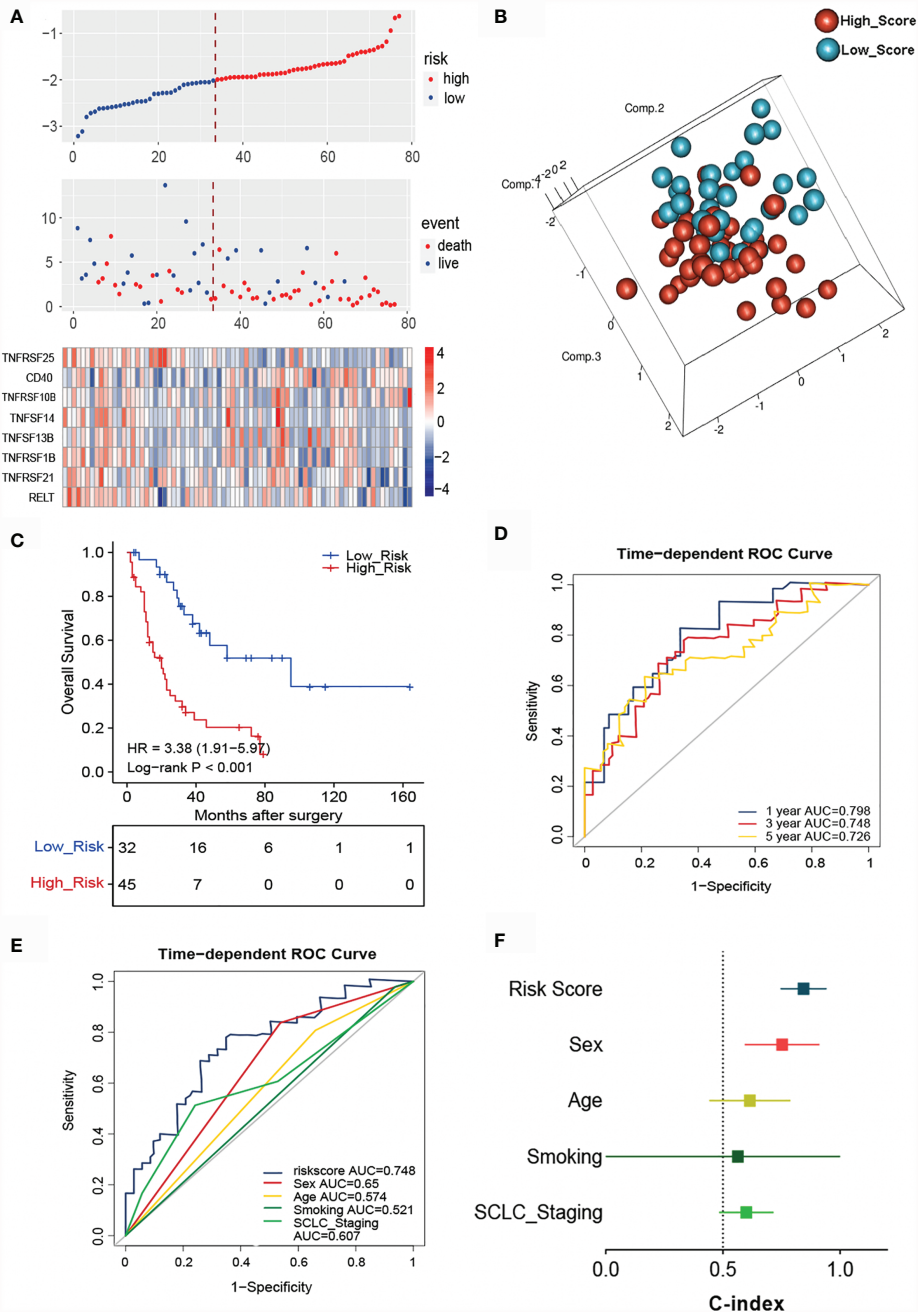
Identification of the TNF family-based signature in SCLCs from the training cohort. **(A)** The distribution of risk score, survival status, and TNF family gene expression profiles. **(B)** PCA of patients with SCLC based on the expression levels of the eight selected genes. **(C)** The Kaplan-Meier curve of OS for patients with SCLC based on the risk score. **(D)** Time-dependent ROC curves of the risk score for 1-, 3-, and 5-year OS prediction for the training cohort. **(E)** ROC analysis for prediction of 3-year survival compared with other clinicopathological factors. **(F)** C-indexes of models based on the risk score and other clinicopathological factors.

The essential role of T cell-related TNF family molecules and their strong predictive value for SCLC inspired us to construct our prognostic model. We developed a TNF-family signature based on the expression levels of eight genes to predict OS for SCLC patients. The risk score was calculated as follows: risk score = (0.3224 × TNFRSF10B) + (0.2429 × CD40) + (0.0316 × TNFSF13B) + (-0.1292 × TNFRSF21) + (-0.2941 × TNFRSF25) + (-0.3208 × TNFRSF1B) + (-0.4752 × RELT) + (-0.5538 × TNFSF14). The risk score for individuals was calculated using this formula, and all patients with SCLC were separated into high- (n=45) and low-risk groups (n=32) using the optimal cutoff point. The distributions of risk scores and gene expression profiles are displayed in **Figure 3A**. PCA indicated obvious between-group heterogeneity (**Figure 3B**). Kaplan-Meier survival curves of OS revealed that high-risk patients had significantly worse OS (HR=3.38, 95% CI: 1.91, 5.97, P<0.001) (**Figure 3C**). We further evaluated the predictive performance of the signature using a time-dependent ROC curve; the AUCs were 0.798, 0.748, 0.72 after 1, 3, and 5 years, respectively (**Figure 3D**). Additionally, the predictive accuracy of the signature for 3-year survival was significantly better than that of several common clinicopathological parameters, including sex, age, smoking history, and cancer stage (**Figure 3E**). The C-index of the signature was as high as 0.845, indicating excellent discriminatory power (**Figure 3F**).

To determine whether the signature could independently predict the OS of patients with SCLC, we conducted univariate and multivariate Cox regression analyses using the training cohort. We confirmed that the risk score functioned as an independent risk factor (HR = 3.734, 95% CI: 1.871, 7.449, P<0.001) compared with other “traditional” clinical parameters like age, sex, smoking history, and tumor stage (**Table 2**).

**Table 2:** Univariate and multivariable Cox regression analysis of TNF family-based signature and outcomes of OS and RFS in training cohort (*N*=77) and validation cohort (*N *= 137).

Variable	Classification	Univariable analysis			Multivariable analysis		
		HR	95% CI	*P* value	HR	95% CI	*P* value
Training cohort (OS)							
Risk score	High or Low	3.908	2.005, 7.617	<0.001	3.734	1.871, 7.449	<0.001
Sex	Male or Female	3.055	1.419, 6.575	0.004	3.080	1.339, 7.087	0.008
Age	≥60 or<60	1.352	0.686, 2.663	0.384	1.307	0.639, 2.677	0.463
Smoking history	Yes or No	2.437	0.334, 17.79	0.38	0.595	0.070, 5.074	0.635
SCLC_staging	IV, III, II, or I	1.353	1.019, 1.797	0.037	1.270	0.958, 1.684	0.097
Validation cohort (OS)							
Risk score	High or Low	3.564	2.086, 6.091	<0.001	3.114	1.793, 5.410	<0.001
Sex	Male or Female	1.192	0.702, 2.025	0.516	1.054	0.521, 2.131	0.884
Age	≥60 or<60	1.432	0.916, 2.24	0.115	1.502	0.941, 2.395	0.088
Smoking history	Yes or No	1.185	0.743, 1.89	0.477	1.048	0.561, 1.958	0.882
SCLC_staging	III, II, or I	1.537	1.161, 2.035	0.003	1.349	1.013, 1.796	0.040
Validation cohort (RFS)							
Risk score	High or Low	3.748	2.272, 6.183	<0.001	3.362	2.020, 5.598	<0.001
Sex	Male or Female	1.549	0.921, 2.604	0.099	1.393	0.721, 2.692	0.324
Age	≥60 or<60	1.083	0.716, 1.637	0.707	1.117	0.725, 1.721	0.616
Smoking history	Yes or No	1.370	0.884, 2.123	0.159	1.064	0.604, 1.875	0.830
SCLC_staging	III, II, or I	1.473	1.131, 1.917	0.004	1.261	0.963, 1.651	0.092

TNF, tumor necrosis factor; HR, Hazard Ratio; CI, Confidence Interval; OS, overall survival; RFS, relapse-free survival.

### Validation of the Signature in a Validation Cohort of SCLC Patients

**Figure 4:**
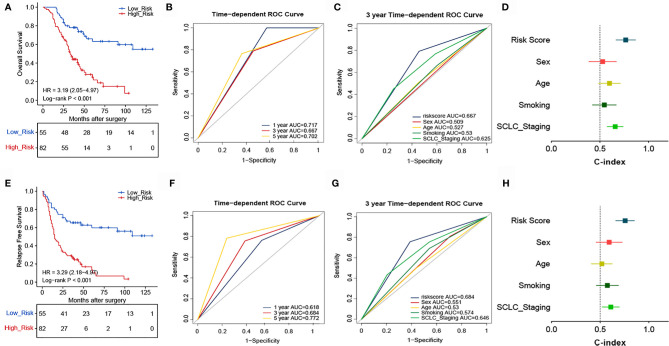
Validation of the prognostic performance of the TNF family based-signature in the validation cohort. **(A, E)** Kaplan-Meier curves of OS and RFS according to risk-score groups in the validation cohort. **(B, F)** ROC curves of the TNF family-based signature for predicting OS and RFS after 1, 3, and 5 years. **(C, G)** ROC curves of the TNF family-based signature for predicting 3-year outcomes of OS and RFS. **(D, H)** C-index for the predictive performance of the signature for OS and RFS.

To confirm whether the TNF family-based signature derived from the training cohort was sufficiently robust for clinical application, we validated the signature in an independent validation cohort. The samples from the validation cohort were obtained from the NCC and consisted of 137 SCLC FFPE samples. We measured the gene expression levels of the FFPE samples using RT-qPCR, after which the patients’ risk scores were calculated, and patients were divided into high- (n=82) and low-risk (n=55) groups according to the optimal risk score cutoff point. As shown in **Figure 4A**, high and low-scoring patients had significantly different outcomes, with high-risk patients more often experiencing an unfavorable OS (HR=3.19, 95% CI: 2.05, 4.97, P<0.001). We also verified the signature’s discrimination and calibration using ROC curves and the C-index (**Figures 4B–D**). The signature exhibited good performance for predicting survival at 1, 3, and 5 years, with AUCs of 0.717, 0.667, and 0.702, respectively. In comparison, sex, age, smoking status, and cancer stage had AUCs of 0.509, 0.527, and 0.625, respectively. Similarly, patients with a low risk score had a longer period of RFS in comparison with those with a high score (HR=3.29, 95% CI: 2.18, 4.97, P<0.001) (**Figure 4E**). Subsequently, ROC and C-index analyses confirmed the robustness of the risk score (**Figures 4F–H**). The multivariate analysis indicated that the risk score was independently correlated with OS (HR=3.114, 95% CI: 1.793, 5.410, P<0.001) and RFS (HR=3.362, 95% CI: 2.020, 5.598, P<0.001). These results confirm that our novel signature is a reliable and effective tool for SCLC prognosis prediction (**Table 2**).

### Evaluation and Validation of the TNF Family-Based Signature in Different Clinical Subsets

To further explore the applicability of the TNF-family-based signature in different clinical settings, we analyzed its predictive efficiency for different clinical subgroups based on age, sex, and smoking status. The results indicated that high-risk patients in nearly all subgroups showed statistically significant worse OS, with the exception of non-smokers (**Figures S2A-F**). As expected, regarding sex, age, and smoking status, the signature remained an independent prognostic biomarker for the prediction of OS and RFS for patients with SCLC in the validation cohort (**Figures S2G-Q**).

### Predicting ACT Benefit in Patients With SCLC

**Figure 5:**
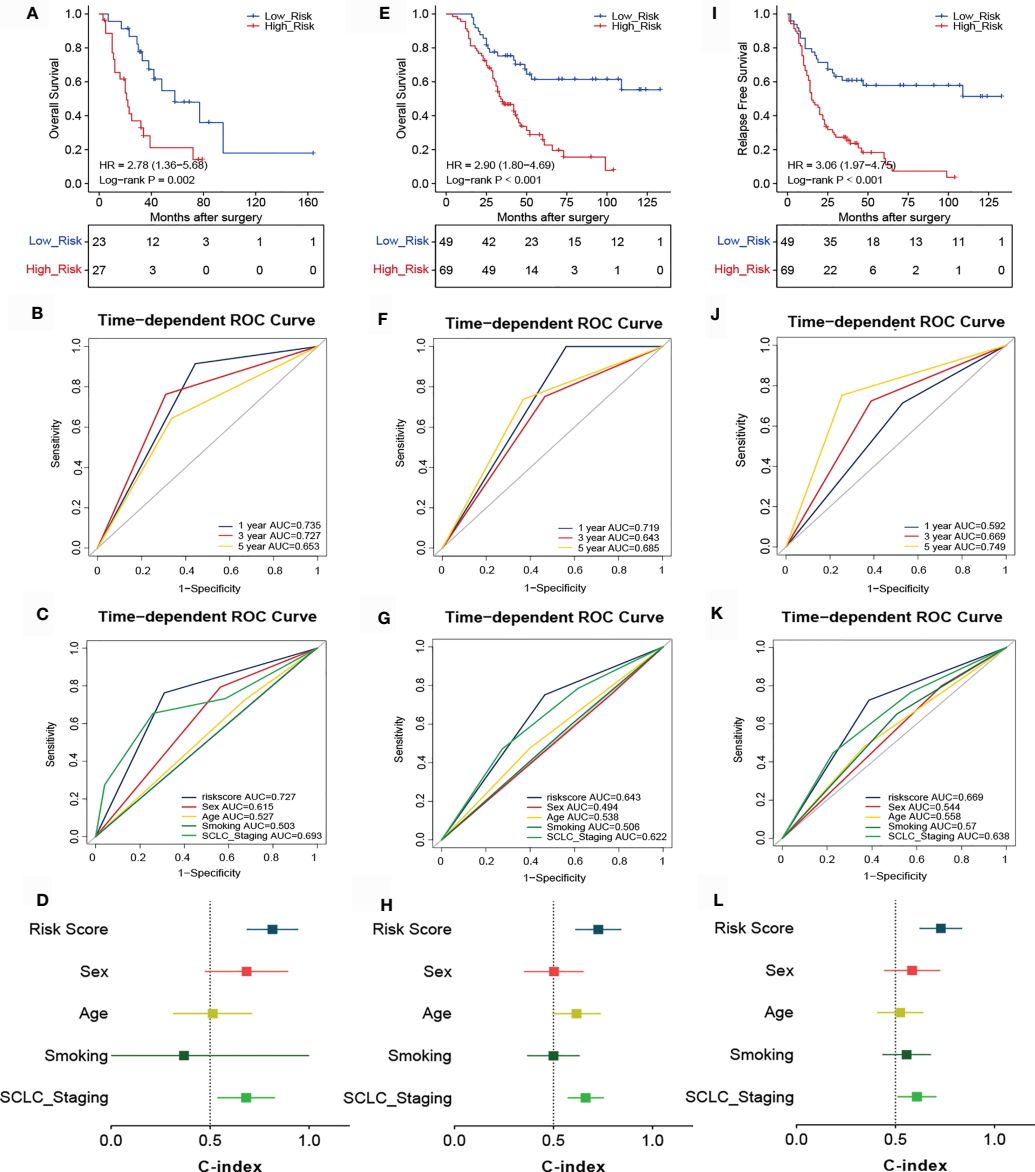
Association between risk score and the survival benefit of adjuvant chemotherapy in patients with SCLC. Kaplan-Meier **(A)** and ROC analyses of OS according to different risk groups in the training cohort **(B, C)**. **(D)** C-indexes for evaluating the performance of the signature for predicting the OS of the training cohort. Kaplan-Meier **(E)** and ROC analyses of OS according to different risk groups in the validation cohort **(F, G)**. **(H)** C-indexes for evaluating the performance of the signature for predicting the OS of the validation cohort. Kaplan-Meier **(I)** and ROC analyses of RFS according to different risk groups in the validation cohort **(J, K)**. **(L)** C-indexes for evaluating the performance of the signature for predicting the RFS of the validation cohort.

Considering that the TIME is an essential factor affecting the efficacy of chemotherapy, we determined whether the risk score could predict the benefit of ACT in patients with SCLC. The association between risk score and chemotherapy benefit was analyzed among patients who received ACT. In the training cohort, patients were classified into high- (n=27) and low-risk (n=23) groups according to the optimal cutoff point of the risk score. There were significant between-group differences in the Kaplan-Meier survival curves, suggesting that the OS of high-risk patients was worse than that of low-risk patients (**Figure 5A**). The ROC analysis showed that the 1-, 3-, and 5-year AUCs were 0.735, 0.727, and 0.653, respectively (**Figure 5B**). These findings indicate that our novel signature demonstrated good accuracy for predicting the prognosis of SCLC patients. The AUC of the risk score (AUC=0.727) was higher than that of other common clinicopathological parameters like sex (AUC=0.615), age (AUC=0.527), smoking status (AUC=0.503), or cancer stage (AUC=0.693) (**Figure 5C**). The novel signature also predicted ACT benefit in the validation cohort. Patients were divided into high- (n=49) and low-risk (n=69) groups, and high-risk patients demonstrated worse OS (**Figure 5E**) and RFS (**Figure 5I**). In addition, the ROC curves for different survival years and various clinical factors also confirmed the predictive value of the signature for ACT response (**Figures 5F, G, J, K**). The C-indexes of the training and validation cohorts also demonstrated the accuracy and discriminatory power of our signature (**Figures 5D, H, L**).

### Biological Pathway and Inflammatory Response Analysis of the TNF Family-Based Signature

**Figure 6:**
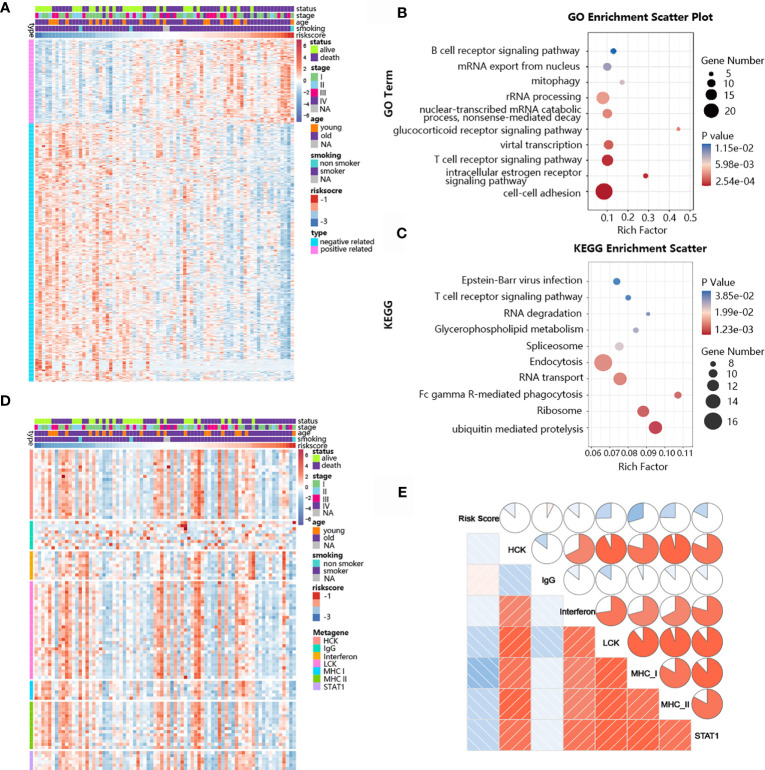
Biological pathways and inflammatory activities of the TNF family-based signature in SCLC from GO and KEGG pathway analyses. **(A)** The most highly associated genes from the TNF family based-signature in SCLC patients from training cohort. **(B)** Gene enrichment with GO terms of the identified genes. **(C)** Gene enrichment with KEGG terms of the identified genes. **(D)** Heatmap of the expression profiles of seven metagenes. **(E)** Correlations between the risk score and seven clusters of metagenes.

The ability of the TNF family-based signature to predict SCLC prognosis inspired our subsequent exploration of signature-related biological pathways. We first selected genes that were closely related to the signature (Pearson |R| > 0.40), and 172 positive and 525 negative genes were filtered out (**Figure 6A**). Next, GO and KEGG analyses were performed to determine the biological roles of the selected genes. The genes in the TNF family-based signature tended to be involved in immune-specific pathways, especially the T cell-related immune response (**Figures 6B, C**). We also determined the association between the risk score and inflammatory activity using a seven-metagene cluster (consisting of genes that are highly correlated and provide robust clustering of samples). The expression patterns and risk scores are presented in **Figure 6D**. In addition, after subjecting the selected genes and metagene cluster to gene set enrichment analysis (GSVA), we found that the risk score was negatively related to LCK, MHC-I, and MHC-II. These results suggest that high-risk patients are more likely to show immunosuppression (**Figure 6E**).

### Immune Cell Infiltration and Immune Checkpoints Associated With the Risk Score

**Figure 7:**
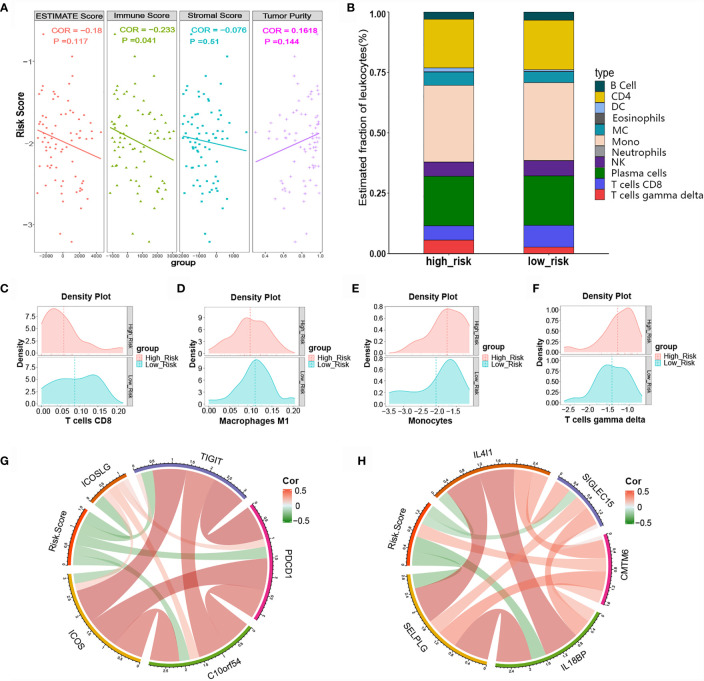
Relationship between the risk score and tumor-infiltrating immune cells as well as immune checkpoints. **(A)** ESTIMATE score, immune score, stromal score, and tumor purity in high- and low-risk patients. **(B)** Difference in tumor-infiltrating immune cells between the high- and low-risk groups. **(C-F)** Difference in immune cell infiltration abundance between the high- and low-risk groups. **(G, H)** Correlation chord chart showing the correlations between risk scores and other immune checkpoint molecules.

Immune cells and the TNF family play vital roles in immune responses and inflammatory reactions. Considering that the immune score and tumor purity have immunotherapeutic implications for cancer patients, we analyzed the relationship between the risk score and immune infiltration status by determining the ESTIMATE score, immune score, stromal score, and tumor purity for the high-risk and low-risk groups. As shown in **Figure 7A**, the high-risk group had lower stromal and immune scores in comparison with the low-risk group, while the former group had greater SCLC tumor purity. We further evaluated the immune infiltration of 22 types of immune cells using CIBERSORT. The relative proportion and composition of immune cells were determined for the high- and low-risk groups of the training cohort, which was stratified using the TNF family based-signature (**Figure 7B**). The density plots showed that the low-risk and high-risk groups had different immune landscapes. For example low-risk patients exhibited significantly greater infiltration of CD8+ T cells (P= 0.0166) (**Figures 7C–F**).

Various novel immune checkpoint molecules have been identified recent years; however, the clinical effects of ICIs, like monoclonal antibodies targeting PD-1 and PD-L1, remain unsatisfactory for SCLC patients. We therefore explored the association between the TNF family based-signature and other classic and novel immunotherapy checkpoint targets. The correlation chord chart showed that the risk score was negatively correlated with the expression levels of immune checkpoints ICOS, ICOSLG, TIGIT, PDCD1, B7-H5, IL4l1, SIGLEC15, IL18BP, and SELPLG (**Figure 7G**). Of note, the risk score was significantly positively correlated with the expression level of CKLF-like MARVEL transmembrane domain-containing 6 (CMTM6), a potential immunotherapy target for high-risk patients (**Figure 7H**).

## Discussion

Due to rapid growth, early metastasis and limited treatment options, SCLC is one of the most lethal tumors. Chemotherapy remains the key backbone of SCLC treatment, and accurate prediction of prognosis and treatment response would surely help to further optimize the choice of personalized treatment and prognosis management for SCLC. Recently, the field of cancer genomics has developed rapidly, and various novel therapeutic targets and prognostic markers have been discovered for multiple types of malignant tumors. Unfortunately, the relative scarcity of tumor tissue samples has limited investigations of potential predictive markers for treatment response that could be applied in clinical settings to patients with SCLC. Immunotherapy has achieved success as a treatment for patients with many different types of tumors, but its efficacy has been limited in SCLC patients (7). Considering the limited knowledge of immune heterogeneity and the anti-tumor status of the TIME of SCLC, exploration of immune mechanisms and TIME regulators are essential for the development of improved treatments for SCLC patients.

The TNF family is expressed in nearly all cells and regulates a wide range of cellular activities, including cell survival, tissue remodeling, and immunity (30). Notably, TNF family members are immunoregulators that play pivotal roles in immune responses, particularly in co-stimulation of T cell responses (31, 32). TNF receptors are implicated in immune surveillance in pancreatic β cell cancer (33). TNF is also present in the TIME of many cancers, where it is thought to enhance cancer growth. TNF produced by myeloid cells was found to promote inflammation-associated tumors (34), and TNF derived from macrophages was implicated in inflammation and subsequent tumor development (35, 36). In addition, TNF in the TIME can cause genetic damage and have other direct effects on malignant cells; for instance, TNF in the TIME can induce tumor cells to undergo the epithelial-mesenchymal transition (EMT), thus facilitating metastasis (37–39). Before these mechanisms were fully appreciated, clinical trials of TNF were initiated, but these trials failed due to severe toxicity (40–43). To move forward with the clinical application of TNF treatment, additional studies are needed to illuminate the roles of TIME-derived TNF in human cancers and its relative importance in tumor prognosis and treatment for early and late stage SCLC patients.

This study was the first retrospective study of SCLC patients to construct an immune-specific signature based on TNF family members for prognosis and treatment benefit prediction. The eight-gene TNF-signature was constructed using a training cohort and verified with qPCR using FFPE specimens from a validation cohort. The signature was an independent prognostic factor for patients with SCLC and showed precise predictive value in several distinct clinical subgroups. Notably, the signature was also useful for predicting chemotherapy response. Our exploration of the association between the signature and immune-inflammatory responses showed that tumor-infiltrating CD8+ T cells were predominant in low-risk patients. Additionally, the risk score was positively associated with other novel immune checkpoints, suggesting that high-risk patients were more likely to benefit from ICI-based therapies.

TNF family members are promising therapeutic targets for reversing tumor immunosuppression, enhancing host antitumor immune response, and improving clinical outcomes for patients with SCLC (44). To better understand the TNF/TNFR family profiles of SCLC patients, 19 ligands and 29 receptors were selected and analyzed in our study. We compared the TNF profiles of SCLC samples and adjacent tissues, and we found that TNF family members were expressed at significantly lower levels in tumor tissues, suggesting that significant heterogeneity exists between tumor tissue and normal tissues. Based on this heterogeneity, a TNF family-based prognostic predictive signature was constructed, including TNFRSF25, CD40, TNFRSF10B, TNFSF14, TNFSF13B, TNFRSF1B, TNFRSF21, and RELT, and the signature was shown to possess excellent predictive value for predicting the prognosis of SCLC patients.

TNFRSF25 (DR3), a cell surface receptor, is mainly expressed by T cells, for which it mediates apoptotic signaling and differentiation (45). TNFRSF25 also enhances the CD4+ T cell response and promotes CD8+ T cell responses and antitumor immunity (46). TNFRSF1B (CD120b) is a membrane receptor that binds TNFα. TNFα is a proinflammatory cytokine that is synthesized by activated macrophages (47). Altered gene and/or protein expression of TNFRSF1B may be a prognostic biomarker for patients with NSCLC (48). CD40, also known as TNFRSF5,regulates cellular and humoral immunity, but the specific physiological functions of CD40 are dependent on cell type and the microenvironment. CD40 may be an effective prognostic marker for lung cancer (49). TNFRSF10B (DR5) is primarily localized on the plasma membrane, where it functions as an apoptotic receptor to induce cell apoptosis. A DR5-targeting antibody has been developed and tested in clinical trials as a therapeutic intervention for cancer patients (50, 51). TNFSF14 (LIGHT, HVEML, CD258), a secreted protein, is a highly effective stimulator of antitumor immune responses, which also influences the plasticity of the TIME (52). TNFSF14 binds the decoy receptor TNFRSF14 to deliver costimulatory signals to T cells (53). TNFSF14 is a promising cancer immunotherapy target that appears to moderate survival and apoptosis in lymphocytes and tumor cells (52). TNFSF13B (BAFF, CD257) is a cytokine with three receptors: BAFF-R, TACI, and BCMA (54). BAFF may influence the maturation, proliferation, and class switching of B cells (55). A high expression level of BAFF may lead to increased lung inflammation and worsened alveolar wall destruction (56). TNFRSF21 (DR6) is an extensively post-translationally modified type I transmembrane protein that acts as a death domain-containing receptor (57). DR6 is expressed ubiquitously in most human tissue types, and particularly in the heart, brain, pancreas, lymphoid organs, as well as by non-lymphoid cancer cell lines (58). High expression of DR6 is associated with elevated levels of anti-apoptosis molecules, and it contributes to tumor cell survival and immune evasion (59). TNFRSF19L (RELT), a type I transmembrane glycoprotein, predominates in immune cells and lymphoid tissues (60). High expression of RELT in epithelial cells induces cell death (61), and RELT-knockdown mice demonstrated negative regulation of the early T cell response phase (62). These findings illustrate that high RELT expression down-regulates immune responses.

After exploring the functions of the eight genes comprising the TNF signature, we verified its robustness by validating it using a large SCLC cohort with FFPE specimens and clinical subgroups. The results of the validation analysis confirmed the high predictive value and accuracy of the TNF family-based signature. These findings inspired us to explore the underlying mechanisms of action and elucidate the role of these genes in SCLC biology. Functional annotation of TNFSF/TNFRSF genes revealed that the signature was closely associated with immune-related processes. Considering the role of the TIME, the signature-related immune landscape was also investigated. Noticeably, we found that low-risk patients showed significantly more infiltrated CD8+ T cells, which are crucial for antitumor immunity (63). The correlations between the signature and seven metagenes were analyzed to provide additional insight into the mechanisms underlying inflammation and immune responses in SCLC patients (64). The risk score was negatively associated with monocyte/myeloid lineage-specific functions and the antigen-presenting process of T cells, thereby revealing the immunosuppressed status of high-risk patients.

In addition, the TNF family-based signature was confirmed as an independent prognostic indicator that effectively stratified patients into high- and low-risk groups with distinct prognosis and survival. Low-risk patients had more favorable outcomes than their high-risk counterparts, largely because they had a greater number of tumor-infiltrating CD8+ T cells. In agreement with our results, CD8+ T cell infiltration has been shown to be an independent positive prognostic factor that is strongly associated with better outcomes in patients with lung cancer (65), colorectal cancer (66), esophageal cancer (67), and other cancers. Noticeably, our signature can be used to predict the ACT response of SCLC patients; low-risk patients derived a greater benefit from ACT. These results were slightly consistent with prior studies (68, 69). The proportion of tumor-infiltrating CD8+ T cells was statistically higher in the low-risk group. Previously published evidence suggests that tumor-infiltrating cells have profound effects on chemotherapy efficacy against various tumors (70). CD8+ T cells are important tumor-infiltrating lymphocytes that play a vital role in antitumor immunity (71). Thus, chemotherapy may induce antitumor immunity by triggering tumor-infiltrating immune cells (72).

ICIs have shown therapeutic potential in several malignancies, including SCLC (73). The expression levels of immune checkpoints can predict the therapeutic response to immunotherapy. Well-studied biomarkers like PD-L1 and TMB are not sufficient to predict the prognosis of SCLC patients. Therefore, novel immunotherapeutic targets are needed to overcome this limitation, and immune checkpoints are being actively researched (74). Our analysis of the correlations between our TNF family-based signature and classical and novel immune checkpoints revealed that the risk score of SCLC patients was positively associated with the expression level of CMTM6. CMTM6, a member of the CMTM family, is a novel immune checkpoint that is localized at the plasma membrane of various cells. Previous studies have shown that CMTM6 influences the maintenance of cancer stem cells and the EMT (75). In addition, depletion of CMTM6 delays tumor growth, enhances tumor-specific T cell activity and reduces the number of exhausted T cells (75). CMTM6 plays an essential role in maintaining cell surface PD-L1 protein stability by preventing it from undergoing ubiquitin-mediated degradation (76). Therefore, CMTM6 may be a useful immunotherapy, especially for patients with resistance to anti-PD-1/PD-L1 treatments. Interestingly, our study suggested that our signature was positively associated with the expression level of CMTM6, but it had no significant correlation with the expression level of PD-L1. There are several potential explanations for this finding. Firstly, PD-L1 expression is usually low or absent in SCLC (77, 78). In the present study, the PD-L1 expression level of the selected samples was too low to allow correlational analysis with the TNF family-based signature. Secondly, CMTM6 regulates PD-L1 at the protein level instead of the mRNA level, so CMTM6 was not expected to affect PD-L1 mRNA expression. CMTM6 is not a general regulator of protein translation or stability (79). We focused on the transcriptional level in this study, so any effects of CMTM6 at the protein level were outside its scope. Thirdly, the effects of CMTM6 are not limited to PD-L1, and its effects in the TIME may be mediated by other mechanisms. The results described above suggest that CMTM6 may be a target for SCLC immunotherapy, especially for high-risk patients with few therapeutic options.

Several limitations of the study should be considered. Firstly, patients and clinicians mainly selected an ACT approach in real-life clinical practice settings, and no random group assignment scheme was used. Secondly, the predictive capacity of the eight-gene-related signature may not be stable within the TIME because it is a heterogeneous mixture. Finally, the study cases were collected retrospectively, and the predictive value of the signature for ACT requires further validation in large, well-designed, and prospective trials.

In conclusion, our study was the first full-scale study of the expression patterns and clinical relevance of TNF family molecules in SCLC. Our novel TNF family-based signature independently predicted the prognosis of patients with SCLC and was a useful tool for evaluating the survival benefit from ACT. Analysis of similar genetic expression patterns may be the best way to advance prognostication and the development of personalized precision medicine approaches for patients with SCLC.

## Data Availability Statement

The datasets presented in this study can be found in online repositories. The names of the repository/repositories and accession number(s) can be found in the article/supplementary material.

## Ethics Statement

The studies involving human participants were reviewed and approved by National Cancer Center/Cancer Hospital of the Chinese Academy of Medical Sciences. The patients/participants provided their written informed consent to participate in this study. Written informed consent was obtained from the individual(s) for the publication of any potentially identifiable images or data included in this article.

## Author Contributions

NS and JH supervised the project, designed the experiments, edited the manuscript, and led the experiments. ZZ, CZ, and PW conducted the experiments and data analysis. ZZ, PW, and CZ prepared all figures and tables. ZZ and PW drafted the manuscript. CZ, GZ, YL, QZ, LW, and ZY collected clinical samples. All authors contributed to the article and approved the submitted version.

## Funding

This work was supported by the CAMS Innovation Fund for Medical Sciences (2017-I2M-1-005), the National Key R&D Program of China (2016YFC1303201), the National Natural Science Foundation of China (81802299, 81502514), the Fundamental Research Funds for the Central Universities (3332018070), and the National Key Basic Research Development Plan (2018YFC1312105).

## Conflict of Interest

The authors declare that the research was conducted in the absence of any commercial or financial relationships that could be construed as a potential conflict of interest.

## Publisher’s Note

All claims expressed in this article are solely those of the authors and do not necessarily represent those of their affiliated organizations, or those of the publisher, the editors and the reviewers. Any product that may be evaluated in this article, or claim that may be made by its manufacturer, is not guaranteed or endorsed by the publisher.
