# Supplementary material for: Tumor Necrosis Factor Family Member Profile Predicts Prognosis and Adjuvant Chemotherapy Benefit for Patients With Small-Cell Lung Cancer
Source: Front Immunol. 2021 Nov 18;12:745769. doi: 10.3389/fimmu.2021.745769 (PMC8637339; doi:10.3389/fimmu.2021.745769)

**Supplementary Figures**

**Supplementary Figure S1. The relationship between TNF family members.**


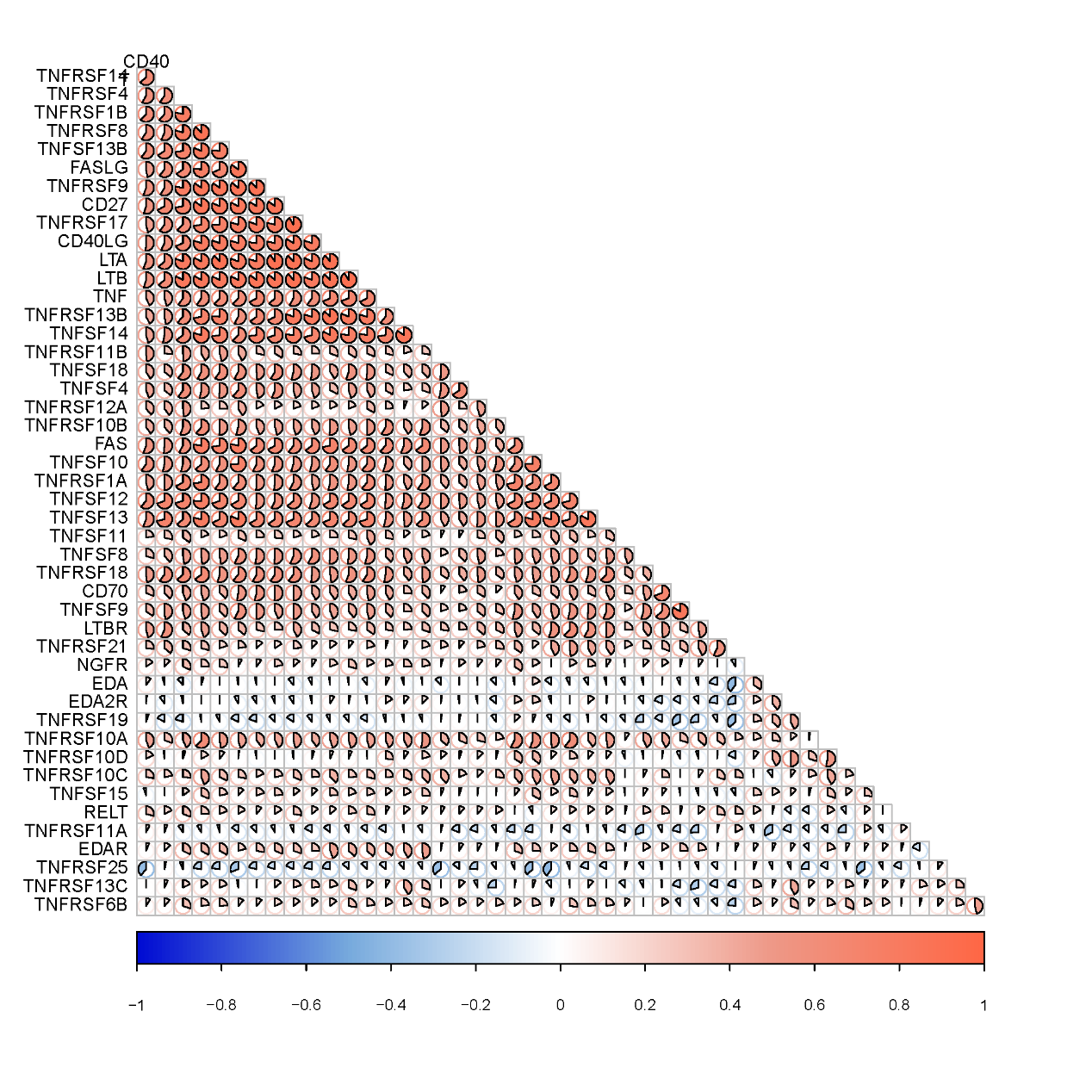


**Supplementary Figure S2. Subgroup analyses of the TNF family based-signature in training and validation cohorts.**

(A-F) Subgroup analysis of TNF based overall survival-related signature in training cohort. (G-L) Subgroup analysis of TNF based overall survival-related signature in testing set. (M-Q) Subgroup analysis of TNF based overall recurrence-free survival-related signature in validation cohort. Patents were divided into different subgroups according to different sex (male and female), age (age ≥ 60 and age<60), and smoking status (smoker or nonsmoker).


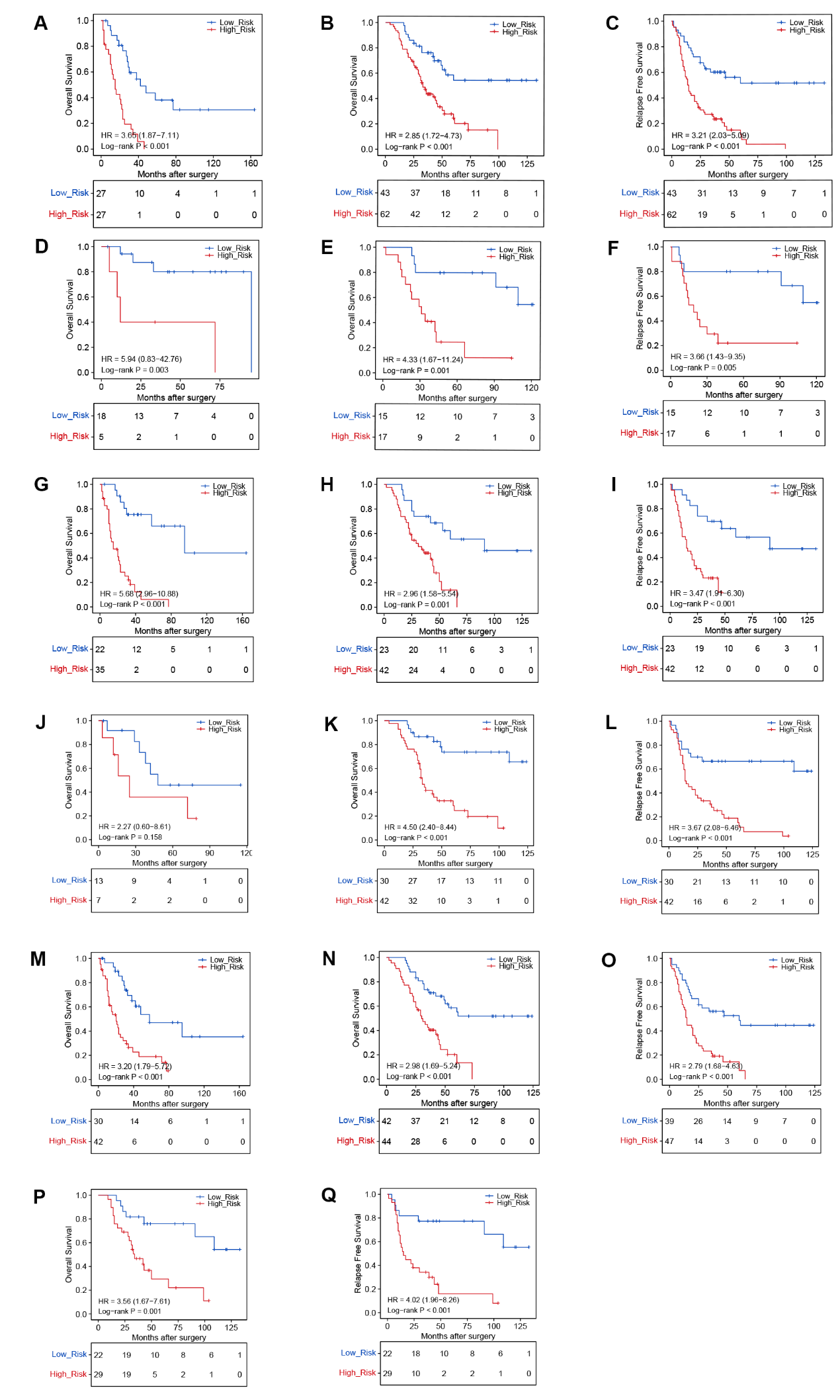

Supplement: Supplementary file 1 [file DataSheet_1.docx]
