# Supplementary material for: Tumor Necrosis Factor Family Member Profile Predicts Prognosis and Adjuvant Chemotherapy Benefit for Patients With Small-Cell Lung Cancer
Source: Front Immunol. 2021 Nov 18;12:745769. doi: 10.3389/fimmu.2021.745769 (PMC8637339; doi:10.3389/fimmu.2021.745769)
Supplement: Supplementary file 2 [file DataSheet_2.docx]

**Supplementary Tables**

**Supplementary Table S1**. Kaplan-Meier curve analysis of TNF family members in training cohort

| **Family** | **Official symbol** | **Aliases** | **HR** | ***P* value** |
| --- | --- | --- | --- | --- |
| TNFSF | CD40LG | TNFSF5, CD154 | 0.5006 | 0.0170 |
| TNFSF | CD70 | TNFSF7, CD27L | 0.5672 | 0.0484 |
| TNFSF | EDA | EDA-A1, EDA-A2 | 1.3511 | 0.3288 |
| TNFSF | FASLG | TNFSF6, CD95-L | 0.7328 | 0.2922 |
| TNFSF | LTA | TNFSF1 | 0.4298 | 0.0152 |
| TNFSF | LTB | TNFSF3 | 0.5259 | 0.0263 |
| TNFSF | TNF | TNFSF2, TNFA | 1.5255 | 0.2510 |
| TNFSF | TNFSF10 | TRAIL, CD253 | 1.7745 | 0.0508 |
| TNFSF | TNFSF11 | RANKL, CD254 | 1.9633 | 0.0287 |
| TNFSF | TNFSF12 | TWEAK | 1.8285 | 0.0598 |
| TNFSF | TNFSF13 | APRIL, CD256 | 0.6988 | 0.2126 |
| TNFSF | TNFSF13B | BAFF, CD257 | 2.4339 | 0.0047 |
| TNFSF | TNFSF14 | LIGHT, HVEML, CD258 | 0.5025 | 0.0221 |
| TNFSF | TNFSF15 | TL1A | 1.5599 | 0.1264 |
| TNFSF | TNFSF18 | GITRL | 0.6054 | 0.0927 |
| TNFSF | TNFSF4 | OX-40L, CD134L, CD252 | 0.4924 | 0.019 |
| TNFSF | TNFSF8 | CD30L, CD153 | 0.7464 | 0.3156 |
| TNFSF | TNFSF9 | 4-1BB-L, CD137L | 0.5016 | 0.0152 |
| TNFRSF | CD27 | TNFRSF7 | 0.5582 | 0.0416 |
| TNFRSF | CD40 | TNFRSF5 | 4.7654 | 0.0191 |
| TNFRSF | EDA2R | TNFRSF27, XEDAR | 1.6678 | 0.1013 |
| TNFRSF | EDAR | EDA-A1R | 0.5964 | 0.1321 |
| TNFRSF | FAS | TNFRSF6, CD95 | 1.7104 | 0.0717 |
| TNFRSF | LTBR | TNFRSF3 | 0.622 | 0.1039 |
| TNFRSF | NGFR | TNFRSF16, CD271 | 0.5864 | 0.1334 |
| TNFRSF | RELT | TNFRSF19L | 0.5031 | 0.0209 |
| TNFRSF | TNFRSF10A | TRAILR1, CD261 | 0.4959 | 0.0651 |
| TNFRSF | TNFRSF10B | TRAILR2, CD262 | 0.3641 | 0.0031 |
| TNFRSF | TNFRSF10C | TRAILR3, CD263 | 1.8155 | 0.0526 |
| TNFRSF | TNFRSF10D | TRAILR4, CD264 | 1.5906 | 0.1118 |
| TNFRSF | TNFRSF11A | RANK, CD265 | 0.502 | 0.0199 |
| TNFRSF | TNFRSF11B | OPG | 1.7612 | 0.1466 |
| TNFRSF | TNFRSF12A | FN14, TWEAKR, CD266 | 1.4739 | 0.2313 |
| TNFRSF | TNFRSF13B | TACI, TNFRSF14B, CD267 | 0.6109 | 0.0874 |
| TNFRSF | TNFRSF13C | BAFFR, CD268 | 0.5752 | 0.0912 |
| TNFRSF | TNFRSF14 | LIGHTR, HVEM, CD270 | 0.607 | 0.0838 |
| TNFRSF | TNFRSF17 | BCMA, TNFRSF13A, CD269 | 1.657 | 0.1576 |
| TNFRSF | TNFRSF18 | GITR, AITR, CD357 | 0.4386 | 0.0093 |
| TNFRSF | TNFRSF19 | TROY, TAJ | 2.0996 | 0.0131 |
| TNFRSF | TNFRSF1A | TNFR1, CD120A | 0.6688 | 0.1614 |
| TNFRSF | TNFRSF1B | TNFR2, CD120B | 0.5582 | 0.0442 |
| TNFRSF | TNFRSF21 | DR6, CD358 | 0.347 | 0.0049 |
| TNFRSF | TNFRSF25 | DR3, TNFRSF12 | 0.3939 | 0.0016 |
| TNFRSF | TNFRSF4 | OX40, CD134 | 0.6078 | 0.0855 |
| TNFRSF | TNFRSF6B | DCR3 | 1.4011 | 0.2521 |
| TNFRSF | TNFRSF8 | CD30 | 0.3584 | 0.0019 |
| TNFRSF | TNFRSF9 | 4-1BB, CD137, ILA | 0.5127 | 0.0323 |

HR, Hazard Ratio.

**Supplementary Table S2.**Primer Sequences for qRT-PCR

| **Gene Name** | **Forward Primer** | **Reverse Primer** |
| --- | --- | --- |
| GAPDH | 5'-GGAGCCAAAAGGGTCATCATCTC-3' | 5'-GAGGGGCCATCCACAGTCTTCT-3' |
| CD40 | 5'-CTGTCCATCCAGAACCACCC-3 | 5'-CTGTGCAGTCACTCACCAGT-3 |
| TNFSF13B | 5'-AGGGGAAGTGCCCTAGAAGA-3 | 5'-GATGTCCCATGGCGTAGGTC-3 |
| TNFRSF21 | 5'-TGAGCATTTGCCCTGGATGA-3 | 5'-AGAGTCCTCGAGCTTTTCCG-3 |
| TNFRSF25 | 5'-CCCATCTGTCACCCTTGGAC-3 | 5'-TGTTACCCACCAACTGGACG-3 |
| TNFRSF1B | 5'-GCTCCACAATGGGAGACACA-3 | 5'-GTCTCCAGCTGTGACCGAAA-3 |
| RELT | 5'-TGGAGGCAGTGGAATCAACC-3 | 5'-TCTCTGTGATCAAGCGCACC-3 |
| TNFSF14 | 5'-CCGCTGTTATGGGAGACTCA-3 | 5'-CAGCTGCACCTTGGAGTAGA-3 |
| TNFRSF10B | 5'-TCGTGAGTATCTTGCAGCCC-3 | 5'-CCAGCAGATGCTCTGACTCC-3 |
